# Medical and Surgical Gynæcology

**Published:** 1892-05

**Authors:** 


MEDICAL AND SURGICAL GYNECOLOGY.—By S. Pozzi, M. D., of Paris. Translated from the French by Brooks H. Wells, M. D., of New York. Volume 2 contains 305 wood engravings and six full-page plates in colors.

This work is more cosmopolitan than those of most French writers, to quote from preface: "I have largely consulted foreign publications; and have quoted as many English, American and German as French names. It is possible I may be criticised for so doing, but 'For whoever thinks there is neither French nor English,' said Voltaire."

This work bears evidence of thought and extensive clinical experience.

He believes in antisepsis, devoting 30 pages to the consideration of the subject in detail.

The author deals with many minor points which adds to the value of the work, while he avoids being prolix on points of little value.

The first volume also treats of anæsthesia, means of wound-closure and the control of hemorrhage, gynecological examinations, metritis, carcinoma of the uterus, displacements of the uterus, deformities of the cervix, artresia, stenosis, atrophy, hypertrophy and disorders of menstruation.

The author and translator have furnished a valuable work, and it deserves extensive sale. R. R. K.

